# Nonlinear pattern of pulmonary tuberculosis among migrants at entry in Kuwait: 1997–2006

**DOI:** 10.1186/1471-2458-8-264

**Published:** 2008-07-30

**Authors:** Saeed Akhtar, Hameed GHH Mohammad

**Affiliations:** 1 Department of Community Medicine and Behavioural Sciences, Faculty of Medicine, Kuwait University, PO Box 24923, Safat 13110, Kuwait; 2 Ports and Borders Health Division, Ministry of Health, PO Box 32830, Rumaithiya 25410, Kuwait

## Abstract

**Background:**

There is a paucity of published data on the pattern of pulmonary tuberculosis among migrant workers entering Middle Eastern countries particularly Kuwait. The objectives of this study were to use routine health surveillance data i) to estimate the prevalence of pulmonary tuberculosis among migrant workers at entry in Kuwait and ii) to determine the occurrence of any time trends in the proportions of pulmonary tuberculosis positive workers over the study period.

**Methods:**

The monthly aggregates of daily number of migrants tested and the number of pulmonary tuberculosis cases detected during routine health examinations of migrant workers from tuberculosis high-prevalence countries were used to generate the monthly series of proportions (per 100,000) of pulmonary tuberculosis cases over 120 months between January 1, 1997 and December 31, 2006 and analysed using time series methods.

**Results:**

The overall prevalence (per 100,000) of documented pulmonary tuberculosis cases among screened migrants was 198 (4608/2328582). Year-specific prevalence (per 100,000) of tuberculosis cases consistently declined from 456 (95% CI: 424 – 490) in 1997 to 124 (95% CI: 110 – 140) in 2002 before showing a steady increase up to 183 (95% CI: 169–197) in 2006. The second-order polynomial regression model revealed significant (*P *< 0.001) initial decline, followed by a significant (*P *< 0.001) increasing trend thereafter in monthly proportions of tuberculosis cases among migrant workers.

**Conclusion:**

The proportions of documented tuberculosis cases among migrant workers showed a significant nonlinear pattern, with an initial decline followed by a significant increasing trend towards the end of the study period. These findings underscore the need to maintain the current policy of migrants' screening for tuberculosis at entry. The public health authorities in Kuwait and perhaps other countries in the region may consider complementing the current screening protocol with interferon-*γ *assays to detect migrants with latent *Mycobacterium tuberculosis *infection. An appropriate curative or preventive chemotherapy of detected tuberculosis cases may help in further minimizing the risk of local transmission of *M. tuberculosis*, while contributing in global efforts to control this public health menace.

## Background

Tuberculosis remains one of the leading infectious causes of death globally, killing nearly 2 million people a year [1]. Sub-Saharan Africa has the highest incidence (290 per 100000), but the most populous countries of Asia have the largest numbers of cases and together account for more than half of the global burden [2]. Tuberculosis control programmes can achieve a high level of treatment success and have been shown to be associated with a decline in reported burden of disease [3-6]. However, for the past two decades, a levelling off or a reverse trend in tuberculosis notifications has been reported from many developed countries [7,8]. This disturbed declining trend has been attributed, in part, to the spread of human immunodeficiency virus, multidrug-resistant  tuberculosis, homelessness, deterioration of living conditions and health care delivery, increased drug abuse, immigration from tuberculosis high to low prevalence countries [7,9,10]. Nonetheless, reasons for this phenomenon are complex, differ from one country to another, and have not been entirely elucidated [11].

Kuwait is a small oil-rich Arabian country in the Persian Gulf region of the Middle East, having a total population of 2.5 million (Kuwaiti: 42%; Non-Kuwaiti 58%), with a gender ratio (male/female) of 1.04 at birth among nationals. Kuwait has a relatively low incidence of tuberculosis with annual notification rate of 24 active tuberculosis cases per 100,000 of population [12]. Resident non-nationals account for about 75% of these active tuberculosis cases per year [12,13], and nearly 1% of these are identified as multidrug-resistant tuberculosis cases [14]. Illegal immigration to Kuwait is almost negligible therefore, seems to play little role in tuberculosis epidemiology. Tuberculosis incidence in Kuwait showed a steady decline from 1965 to 1989. Subsequently, however, there was a rise of 2.3% per year from 1989 to 1999, both among nationals and non-nationals suggestive of *Mycobacterium tuberculosis *transmission from non-nationals to nationals, since a large proportion of migrants from tuberculosis high-burden countries live and work in Kuwaiti homes as domestic workers [12]. Notwithstanding the possibility of *M. tuberculosis *transmission from migrants to Kuwaiti nationals, there is a lack of empirical evidence for such local transmission [15].

The epidemiological importance of migration from tuberculosis high to low incidence countries has been recognized for several years; the main countermeasure has been implementation of screening programs for immigrants at the time of arrival [16,17]. But it not clear that to what an extent the increased immigration from high-incidence countries contributes to an increased risk of tuberculosis in host community of low-incidence countries [18]. Elsewhere immigrants from high-incidence countries to developed and Middle Eastern countries reportedly have high prevalence of tuberculosis [19,20], but there is a paucity of published data on the prevalence of tuberculosis in migrant workers entering Kuwait. Here, we take advantage of the routine screening of migrants for tuberculosis, upon arrival in Kuwait from tuberculosis-endemic regions, to do a first large-scale quantification of the tuberculosis status of this work population. Specifically, the cumulated data on the results of tuberculosis screening of these workers over the past ten years gave us an opportunity in this study not only 1) to estimate the prevalence of tuberculosis in this population of workers, but also 2) to ascertain if any significant time trend or changes had occurred in the prevalence of tuberculosis among these workers during the recent past.

## Methods

### Setting and study population

Migrants constitute about 80% of the labour force in Kuwait, and majority of them usually have a low educational attainment. These migrants originate from tuberculosis high-burden countries predominantly from Southeast Asia, Eastern Mediterranean and African regions wherein prevalence (per 100,000) of tuberculosis ranges from 152 to 547 [21]. There is large turn over of these workers; every year thousands of them leave and new ones arrive in Kuwait. Of the migrants, 46% are 20 to 44 year old and predominantly live as single, mainly because of their inability to fulfil a legal requirement of minimum wages to be able to bring their families [22,23]. Health services are free for all citizens and residents in Kuwait. In public sector, health-care system is made up of six administratively independent health-care sites; each comprises a general hospital, a health center, specialized clinics and dispensaries [24]. In Kuwait, a single tuberculosis control unit and the Kuwait National Central Laboratory under the Ministry of Health are responsible for prevention, diagnosis, treatment, case recording/reporting, contact tracing and treatment supervision under DOTS (Directly Observed Therapy, Short-course) strategy. On diagnosis of tuberculosis, all patients are offered treatment using first-line anti-tuberculosis drugs including isoniazid, rifampicin, ethambutol, and streptomycin based on drug sensitivity pattern [15].

### Data source

Monthly aggregates of test results for diagnosis of pulmonary tuberculosis among migrants entered in Kuwait between January 1, 1997 and December 31, 2006 were available for this study. These migrants predominantly come from India (31%), Bangladesh (14%), Sri-Lanka (14%), Egypt (12%), Indonesia (9%), Philippine (5%), Pakistan (5%) and 10% from other countries including those from African counties such as Tanzania, Mali, Gambia, Sudan (12%) [25,26]. Routine consensual medical examination procedures are conducted on these workers upon their arrival by the Ports and Borders Health Division of the Ministry of Health, Kuwait. For the diagnosis of tuberculosis, migrants were screened by the serial application of various tests. For each migrant chest radiograph was taken. In the presence of any suspicious lesion in the lungs, confirmatory tuberculosis diagnosis was made by sputum smear examination for acid fast bacilli (AFB) using Ziehl Neelsen technique and bacterial culture. Subsequently, migrant worker was classified as a case of tuberculosis if sputum smear and/or bacterial culture were positive for AFB [27].

### Ethics

As noted above, on arrival in Kuwait, migrants were screened for various infections including *M. tuberculosis *before issuance of residency permit. Verbal consent was solicited after fully informing each migrant about the purpose of screening. These procedures were performed according to a stated governmental policy. The study protocol was approved by the Ethics Review Committee of Faculty of Medicine, Kuwait University.

### Statistical methods

The monthly aggregates of daily number of migrants tested and the number of pulmonary tuberculosis cases detected were used to generate the monthly series of proportions of pulmonary tuberculosis cases (per 100,000) over 120 months from January 1, 1997 to December 31, 2006. These monthly proportions (per 100,000) of pulmonary tuberculosis cases among migrants were used for all further analyses unless stated otherwise. Overall and year-specific prevalences (per 100,000) of tuberculosis cases along with their 95% confidence intervals (CI) were calculated.

### Time series analysis

**Figure 1:**
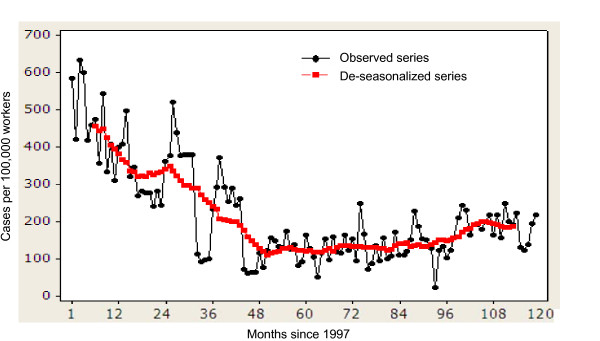
Distribution of proportions of pulmonary tuberculosis cases (per 100, 000) among migrants at entry in Kuwait: 1997–2006.

We employed standard time series methods to assess and model long term trends in the data [28]. Specifically, the purpose of this time series model was to describe any potential temporal trend in the proportions of tuberculosis cases among migrants at entry in Kuwait. We previously demonstrated a significant seasonality in the proportions of tuberculosis cases among migrants [29], therefore, trend estimation was done by first deseasonalizing the series using the moving average smoothing method. We smoothed the data by taking a 13-point (months) moving average filter (Figure 1). The modeling of the trend was then performed following the removal of seasonal effects by initially fitting a locally weighted (Lowess) scatterplot smoother (with bandwidth = 0.3) to explore the form of the long-term trend in the relationship between time (months) and monthly proportions of pulmonary tuberculosis cases [30]. Examination of the results from this exercise suggested the existence of a possible nonlinear temporal trend, and therefore a polynomial regression model was fitted to the deseasonalized data to model the observed monthly proportions of tuberculosis cases with respect to "time", and a quadratic term of time (i.e. time^2^). The goodness-of-fit of the final model was evaluated via residual analysis by plotting residuals against fitted values and also versus the time variable [31].

## Results

### Descriptive statistics

During 120 months from January 1, 1997 to December 31, 2006, 2328582 migrant workers from pulmonary tuberculosis high-prevalence countries entered Kuwait and were eligible for tuberculosis screening. The mean (± standard deviation) number of migrants screened for tuberculosis each month were 19405 ± 7253. The overall prevalence (per 100,000) of documented pulmonary tuberculosis cases among migrants was 198 (4608/2328582). Total yearly pulmonary tuberculosis cases (per 100,000) consistently declined from 456 (95% CI: 424 – 490) in 1997 to 124 (95% CI: 110 – 140) in 2002 before it showed a yearly increase up to 184 (95% CI: 171–199) and 183 (95% CI: 169–197) cases in 2005 and 2006 respectively (Table 1).

**Table 1:** Distribution of proportions (per 100,000) of pulmonary tuberculosis cases among migrants at entry screening in Kuwait: 1997–2006.

Year	Total tested	No. positive	No. positive (per 100,000)	95% confidence limits
1997	161682	737	456	424 – 490
1998	163326	508	311	285 – 339
1999	177129	523	295	271 – 322
2000	130984	261	199	176 – 225
2001	178472	225	126	111 – 144
2002	221566	275	124	110 – 140
2003	254608	334	131	118 – 146
2004	327216	436	133	123 – 144
2005	356983	657	184	171 – 199
2006	356616	652	183	169 – 197
				
Total	2328582	4608	198	192 – 204

### Polynomial regression model

**Figure 2:**
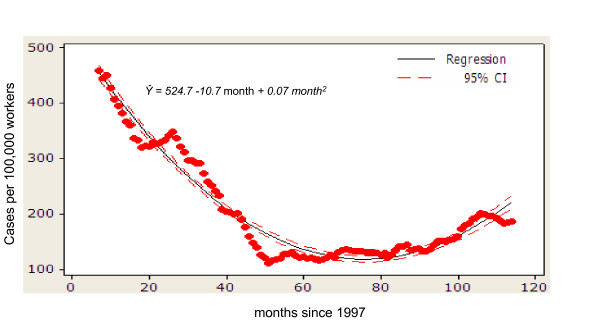
Polynomial regression model fitted to deseasonalized data on the proportions of pulmonary tuberculosis cases among migrants at entry in Kuwait: 1997–2006.

Overall second-order polynomial regression model with time as the single predictor was significant (*F*-statistic = 961; *p *< 0.001) (Table 2). The polynomial terms in the model were also statistically significant (*p *< 0.001), and the point estimates (± standard errors) were ${\hat{\beta }}_{0}$ = 524.684 (± 7.853), ${\hat{\beta }}_{1}$ = -10.657 (± 0.294), ${\hat{\beta }}_{2}$ = 0.070 (± 0.002). The monthly series of proportions of pulmonary tuberculosis cases among migrants revealed a significant (*P *< 0.001) initial decline, followed by a significant (*P *< 0.001) increasing trend thereafter during 120 months of the study period (Figure 2). The two terms in the model together explained about 95% variation in the monthly proportions of tuberculosis cases among migrants (coefficient of determination: *R*^2 ^= 0.948). The plot of observed verses predicted monthly proportions of tuberculosis cases showed adequate fit of the model. Residual analysis to evaluate the aptness of the model suggested that quadratic response function is a good-fit.

**Table 2:** Polynomial regression model of the deseasonalized monthly proportions (per 100,000) of pulmonary tuberculosis cases among migrants at entry screening in Kuwait, 1997–2006.

*Linear and quadratic terms*	*Un-standardized partial regression coefficients*		*t-statistic*	*p*
			
	*Estimate*	*SE*		
Time (${\hat{\beta }}_{1}$)	-10.657	0.294	36.25	< 0.001
Time^2 ^(${\hat{\beta }}_{2}$)	0.070	0.002	29.78	< 0.001
Constant (${\hat{\beta }}_{0}$)	524.684	7.853		

SE = standard error

Coefficient of determination (R^2^) = 0.948

*F*-statistic for overall significance of the model = 961; *P *< 0.001

## Discussion

To our knowledge, this study constitutes one of the largest ever investigations conducted any where in the world for estimating the tuberculosis burden in migrants at entry to tuberculosis low-incidence regions. There is a limited evidence to suggest that migrants from tuberculosis high-burden countries pose a threat to low-incidence host communities [32,33]. However, it has been argued that migrants with latent *M. tuberculosis *infection may remain undetected thus posing a threat at least within their migrant communities, since many migrants are socially isolated and live in overcrowded conditions known to enhance the spread of infection [34,35]. Moreover, this topic is of particular relevance to the countries in Middle East. Kuwait like other countries in the region has a major influx of migrants from tuberculosis high-burden areas. Many of these migrants serve as domestic workers and live in Kuwaiti homes – a pattern of social mixing with host communities perhaps unreported from other tuberculosis low-incidence countries. These migrants thus, may serve as sources of new *M. tuberculosis *infection not only to Kuwaiti nationals but also to migrant community in Kuwait [12]. Also, it has been suggested that screening for tuberculosis and infection with *M. tuberculosis *among migrants has the potential to yield a large number of persons who can benefit from curative or preventive interventions [36].

In Kuwait, we found that the overall prevalence (per 100,000) of tuberculosis cases among migrants was 198 (4608/2328582) or 0.198% during the entire 10-year study period. Almost a similar magnitude of prevalence of tuberculosis has been reported in high-burden counties in South Asia [37,38]. The longitudinal data series based on a 10 year period of observations also uniquely allowed an investigation of the temporal epidemiology of tuberculosis in workers migrating to the country. This has also specifically enabled us to establish that the proportions of migrants with pulmonary tuberculosis at entry in the country have reduced dramatically over the past decade, such that the tuberculosis prevalence in the cohort of workers recruited in 2002 was only around 0.124% (275/221566) in contrast to a peak of 0.456% (737/161682) observed in counterparts in 1997. However, in the following years, a small but significant reversal in the prevalence of tuberculosis cases among migrants occurred, which needs to be monitored.

Trend analysis of these data revealed the occurrence of a nonlinear pattern in the prevalence of tuberculosis in migrants over the 10-year study period. Proportions of tuberculosis cases among workers showed an initial decline between 1997 and 2002 and a subsequent steady increase till the end of the study. The observed initial downward trend in the proportions of tuberculosis cases appears to corroborate previous findings of decreasing prevalence of tuberculosis during the same period in migrants from India, other Asian countries and sub-Saharan Africa to Canada [39]. Also decreasing prevalence of tuberculosis among migrants in our study tended to mimic decreasing tuberculosis burden in Southeast Asia and Eastern Mediterranean region during the same period [40]. This observed decline in the prevalence of tuberculosis over 1997 to 2002 may be the result of an effective implementation of WHO-recommended DOTS strategy during the same period by the public health authorities in the respective countries of origin of these migrants [40,41]. If found to be true, this suggests that sustained DOTS intervention in affected areas over several years could by reducing transmission in those areas contributed significantly in minimizing the risk of exporting *M. tuberculosis *infection into Kuwait and perhaps to other countries in the region. Alternatively, this decline may simply indicate that more workers from a different socio-economic background with lower prevalence of tuberculosis were enlisted during that period. We do not have pertinent data at present to investigate this likely change in migrants' demographic characteristics which might have been associated with the observed decline and this aspect merits further investigations.

The slight but significant increase in proportions of tuberculosis cases among migrants towards the end of the time series (2005–2006) was consistent with contemporary reports of increased global tuberculosis caseloads [21,42], and with the projected increase in the tuberculosis burden during the same period for the countries of origins of the migrants from Southeast Asia, Eastern Mediterranean and African regions [21,40]. A longer period of observation however is required to confirm this small but significantly increasing trend in the prevalence of tuberculosis at the end of time series. This upward trend towards the end of the study period may be an outcome of a shift in the health priority of public health authorities in the endemic countries resulting in the slow down of tuberculosis control efforts. Alternatively, this increasing trend may be a mirror image of increasing burden of multidrug-resistant tuberculosis in countries of origins of the migrants as suggested previously [21,43,44]. We do not have relevant data to corroborate this contention. However, as noted earlier, about 75% of 500 tuberculosis cases in Kuwait each year occur among migrants. Of these tuberculosis cases, 1% are multidrug-resistant tuberculosis cases and nearly all of them occur in resident migrants [12-14].

### Limitations of the study

Some limitations of this study should be considered while interpreting the results. First, as only few variables of interest were available for longitudinal analysis, we are unable to evaluate the roles of demographic factors, *e.g*. age, gender, for their potential associations with the observed changes in the prevalence of tuberculosis among migrants. Second, the non-availability of information on exact locations within their countries of origins precluded any spatial or location-based analysis in this study. Finally, some workers might have been incubating *M. tuberculosis *infection and/or at early stage of the disease and remained undetected with current screening protocol. It is therefore, likely that the proportions of migrants with pulmonary tuberculosis may have been some what underestimated in this study.

## Conclusion

Analysis of the longitudinal screening data on pulmonary tuberculosis has shown not only that the prevalence of pumonary tuberculosis may be declining in the migrants thus reducing the risk that they may pose to the nationals and resident migrants' community in the host country but also that tuberculosis control in endemic countries may be a contributory factor and indeed should be maintained to keep the incidence of *M. tuberculosis *infection declining. The final conclusion of specific significance to public health authorities in Kuwaiti and other Gulf countries' is that the data, particularly either the levelling off or slight rise in tuberculosis in these migrants towards the end of the study period, suggest that there is a need to maintain the current policy of entry screening, which has facilitated the control of tuberculosis so far. However, this strategy appears to be inadequate for detection of migrants with latent *M. tuberculosis *infection, since, as noted earlier each year 75% of about 500 new cases of tuberculosis are notified among the resident migrants in Kuwait [12,13]. Therefore, to detect migrants with latent infection the current screening protocol may be complemented with more sensitive techniques such as interferon-*γ *assays reportedly having estimated sensitivity: 80–95% and specificity: 95–100% to detect latent *M. tuberculosis *infection [45]. The more sensitive screening protocol combined with treatment of detected cases may ensure the maintenance of minimum risk of local transmission of *M. tuberculosis *and contribute in global efforts to control this public health menace.

## Competing interests

The authors declare that they have no competing interests.

## Authors' contributions

SA conceived, designed, analyzed, interpreted the data and drafted the manuscript. HGHHM supervised data collection and reviewed the manuscript. Both the authors have read and approved the final manuscript.

## Pre-publication history

The pre-publication history for this paper can be accessed here:


